# Mechanism of Salt Tolerance and Plant Growth Promotion in *Priestia megaterium* ZS-3 Revealed by Cellular Metabolism and Whole-Genome Studies

**DOI:** 10.3390/ijms242115751

**Published:** 2023-10-30

**Authors:** Lina Shi, Xiaoxia Zhu, Ting Qian, Jiazhou Du, Yuanyuan Du, Jianren Ye

**Affiliations:** 1 Co-Innovation Center for Sustainable Forestry in Southern China, College of Forestry, Nanjing Forestry University, Nanjing 210037, China; sln@njfu.edu.cn (L.S.); xiaoxiazhu@njfu.edu.cn (X.Z.); qt_njfu@163.com (T.Q.); dujiazhou@njfu.edu.cn (J.D.); duyuanyuan010622@163.com (Y.D.); 2 Jiangsu Key Laboratory for Prevention and Management of Invasive Species, Nanjing Forestry University, Nanjing 210037, China

**Keywords:** plant-growth-promoting rhizobacteria (PGPR), compatible substance, γ-aminobutyric acid (GABA), polyamines, rhizosphere colonisation, soil enzyme activity

## Abstract

Approximately one-third of agricultural land worldwide is affected by salinity, which limits the productivity and sustainability of crop ecosystems. Plant-growth-promoting rhizobacteria (PGPR) are a potential solution to this problem, as PGPR increases crop yield through improving soil fertility and stress resistance. Previous studies have shown that *Priestia megaterium* ZS-3(ZS-3) can effectively help plants tolerate salinity stress. However, how ZS-3 regulates its metabolic adaptations in saline environments remains unclear. In this study, we monitored the metabolic rearrangement of compatibilisers in ZS-3 and combined the findings with genomic data to reveal how ZS-3 survives in stressful environments, induces plant growth, and tolerates stress. The results showed that ZS-3 tolerated salinity levels up to 9%. In addition, glutamate and trehalose help ZS-3 adapt to osmotic stress under low NaCl stress, whereas proline, K^+^, and extracellular polysaccharides regulate the osmotic responses of ZS-3 exposed to high salt stress. Potting experiments showed that applying the ZS-3 strain in saline and neutral soils could effectively increase the activities of soil acid phosphatase, urease, and invertase in both soils, thus improving soil fertility and promoting plant growth. In addition, strain ZS-3-GFP colonised the rhizosphere and leaves of *Cinnamomum camphora* well, as confirmed by confocal microscopy and resistance plate count analysis. Genomic studies and in vitro experiments have shown that ZS-3 exhibits a variety of beneficial traits, including plant-promoting, antagonistic, and other related traits (such as resistance to saline and heavy metal stress/tolerance, amino acid synthesis and transport, volatile compound synthesis, micronutrient utilisation, and phytohormone biosynthesis/regulatory potential). The results support that ZS-3 can induce plant tolerance to abiotic stresses. These data provide important clues to further reveal the interactions between plants and microbiomes, as well as the mechanisms by which micro-organisms control plant health.

## 1. Introduction

Salinity has become a major contributor to land degradation and the decline in plant productivity, posing a serious threat to sustainable agricultural production and global food security [1]. The rhizosphere microbiome, which is known as the second genome of plants, is essential for the plant immune system and regulating various processes related to plant growth and development [2,3]. Ample evidence has revealed that beneficial plant micro-organisms can improve the adaptation of plants to salinity [4,5].

Bacterial survival in challenging high-salt environments is possible [6]. Bacteria adapt to high salt concentration stress by different methods. One of the most common strategies is the accumulation of compatible substances, such as sugars, amino acids (aa), and glycine betaine [7,8]. These molecules act as compatible substances and protect cells from damage while maintaining normal cellular functions [9]. Exopolysaccharides (EPS) also provide a protective mechanism and play a crucial role in nutrient uptake, aggregation, adhesion to the plant root surface, and biofilm formation [10,11]. In addition, gram-negative bacteria alter membrane composition by changing the fatty acid saturation or phospholipid composition to better contend with changes in swelling stress [12].

*Priestia megaterium* is a typical gram-positive strain with numerous patents and industrial applications in biotechnology [13]. In agriculture, the great potential of *P. megaterium* for plant growth promotion (PGP) and biotic/abiotic control has been characterised, as repeatedly demonstrated in different crops [14,15,16]. Previous work has shown that *P. megaterium* ZS-3 (ZS-3) improves plant growth under salt stress by adjusting the root architecture, promoting photosynthesis, alleviating oxidative and osmotic stress, and inducing systemic resistance [17]. However, the effects of salt stress on strain ZS-3 metabolism and the strategies utilised by ZS-3 for the adaptation to salt stress are unclear.

The ongoing exploration of whole-genome sequences of individual strains, as well as gene function prediction and characterisation, will unveil new frontiers for understanding how bacterial species thrive in different environments, as well as for understanding their physiology and evolution in general. Recently, researchers have elucidated the genomes of several *Priestia* strains to determine genome-scale metabolic models for industrial micro-organisms [18,19], elucidate the phylogeny of the genus *Bacillus* [20], investigate the *Priestia* strain’s ability to biotransform soil contaminants [21,22], or determine the utility of strains as efficient expression hosts [23]. In this study, we attempted to explore how strain ZS-3 survives in stressful environments and can help plants grow and resist disease. Therefore, we monitored the metabolic changes in strain ZS-3 and analysed the salt tolerance, growth promotion, and disease resistance properties of strain ZS-3 based on genomic data. Further supporting evidence was provided for the potential of strain ZS-3 to promote plant growth under stressful environments.

## 2. Results

### 2.1. Salt-Tolerant Growth Characteristics of P. megaterium ZS-3

**Figure 1:**
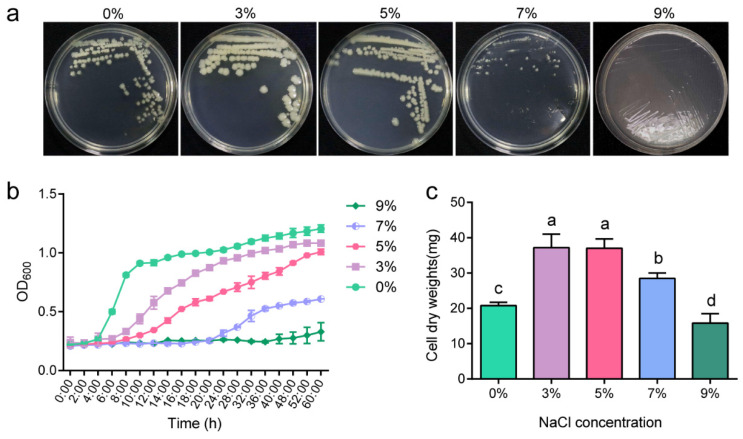
*P*. *megaterium* ZS-3 is a moderately salt-tolerant strain. (**a**) ZS-3 was inoculated on LB plates supplemented with 0%, 3%, 5%, 7%, or 9% NaCl. Images of strain ZS-3 colony formation were recorded at 36 h. (**b**) Growth curves for *P*. *megaterium* ZS-3 generated from Bioscreen C were monitored for 60 h at 28 °C. OD_600_ measurements were collected automatically every 2 h. Values represent the means of three biological experiments, each in ten technical replicates. (**c**) The cell dry weight of ZS-3 at different salt concentrations was quantified at 72 h. Values represent the means of three biological experiments, each in three technical replicates. Error bars represent SE. Different lowercase letters above the bars represent significant differences based on one-way ANOVA (*p* < 0.05).

To study the salt tolerance of strain ZS-3, we evaluated the growth of the strain on solid and liquid media at different salt concentrations. Strain ZS-3 was a moderately salt-tolerant bacterium, as it showed colony formation on LB plates supplemented with 0–9% NaCl (Figure 1a). When the salt concentration was 3%, ZS-3 entered the logarithmic growth period after 28 h of incubation (Figure 1b). When the salt concentration was 9%, no logarithmic growth period was detected, and growth was severely inhibited (Figure 1b). In addition, we examined the cell dry weight of ZS-3 at different salt concentrations (Figure 1c). The cell dry weight of ZS-3 was increased by 78.97%, 78.17%, and 37.08% in the 3%, 5%, and 7% treatment groups, respectively, compared to the 0% group. However, the cell dry weight of ZS-3 in the 9% treatment group was significantly lower than that in the 0% treatment group. The increase in salt concentration caused a growth lag in strain ZS-3.

### 2.2. High Salt Causes P. megaterium ZS-3 Metabolites to Accumulate

**Figure 2:**
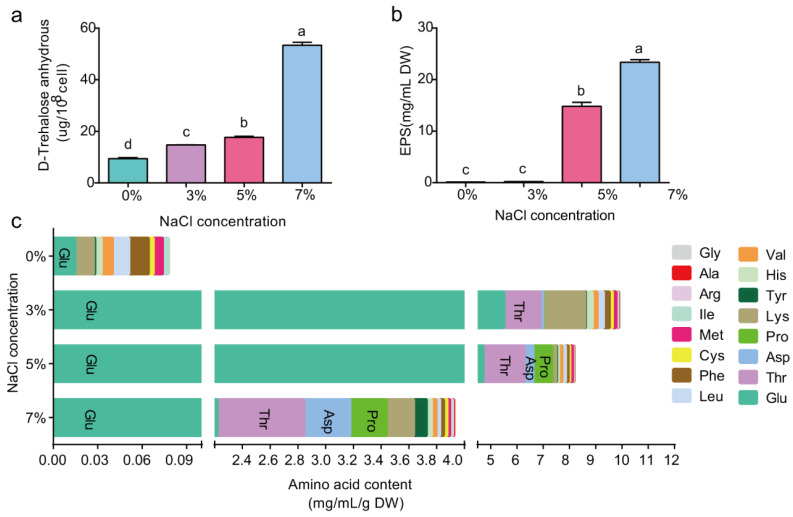
The intracellular D-trehalose (**a**), aa (**b**) and EPS (**c**) in *P. megaterium* ZS-3 were quantified at different salt concentrations (0%, 3%, 5%, and 7% NaCl). Values represent the means of three biological experiments, each in three technical replicates. Error bars represent SE. Different lowercase letters above the bars represent significant differences based on one-way ANOVA (*p* < 0.05).

Since a devastating effect on ZS-3 growth was observed with 9% NaCl stress, trehalose and aa levels were measured at 0–7% NaCl only. The trehalose content increased by 56.24%, 87.43, and 466.67% in the 3%, 5%, and 7% treatment groups, respectively, compared to the 0% treatment group (Figure 2a). Similarly, the type and concentration of aa in ZS-3 cells changed with increasing salt concentration (Figure 2b). Of all the aas, the fluctuations in the glutamate (glu) content were the most pronounced. Compared to the 0% treatment, the glu content increased 374-, 320-, and 150-fold under the 3%, 5%, and 7% treatments, respectively. Ten aas were detected in the 0% treatment group. The abundances of threonine, aspartic acid, proline (pro), alanine, glycine, and arginine were measured in only the 3%, 5%, and 7% treatments.

EPS production by plant-growth-promoting rhizobacteria (PGPR) is often reported as a growth strategy that forms a protective film around cells to protect the cells from external influences in the presence of biotic and abiotic stresses. Therefore, EPS production by ZS-3 under salt stress was assessed. As shown in Figure 2c, strain ZS-3 secreted EPS at different salt concentrations, and 95- and 150-fold increases in EPS production were observed in the 5% and 7% treatments, respectively, compared to the 0% treatment. No significant differences were observed in the 3% treatment for EPS production.

### 2.3. Ion Content In Vivo and In Vitro

**Figure 3:**
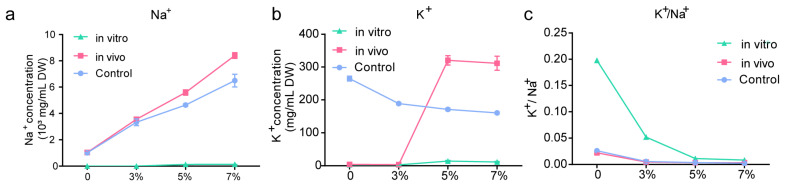
Na^+^ and K^+^ concentrations of *P. megaterium* ZS-3 in the presence of different salt concentrations: (**a**) Na^+^ concentration; (**b**) K^+^ concentration; and (**c**) K^+^/Na^+^. ZS-3 was grown in different media for 48 h, and then the supernatant was collected by centrifugation to determine the ion concentration in vitro. The precipitated cells were broken by ultrasound and centrifuged again, and the supernatant was used to determine the ion concentrations in vivo. LB medium without bacterial inoculum was used as a control. Values represent the means of three biological experiments, each in three technical replicates. Error bars represent SE.

As shown in Figure 3a, the Na^+^ concentration was significantly higher in the 3%, 5%, and 7% treatment groups than in the 0% treatment group. This indicates that ZS-3 converts and absorbs Na^+^ from the environment, which is dependent on high accumulation of intracellular K^+^ (synchronisation). In vivo K^+^ concentration was significantly higher in the 5% and 7% treatment groups compared to the 0% treatment group (Figure 3b); therefore, the in vivo K^+^/Na^+^ balance was maintained (Figure 3c).

### 2.4. General Characteristics of the Whole-Genome Sequence of P. megaterium ZS-3

**Figure 4:**
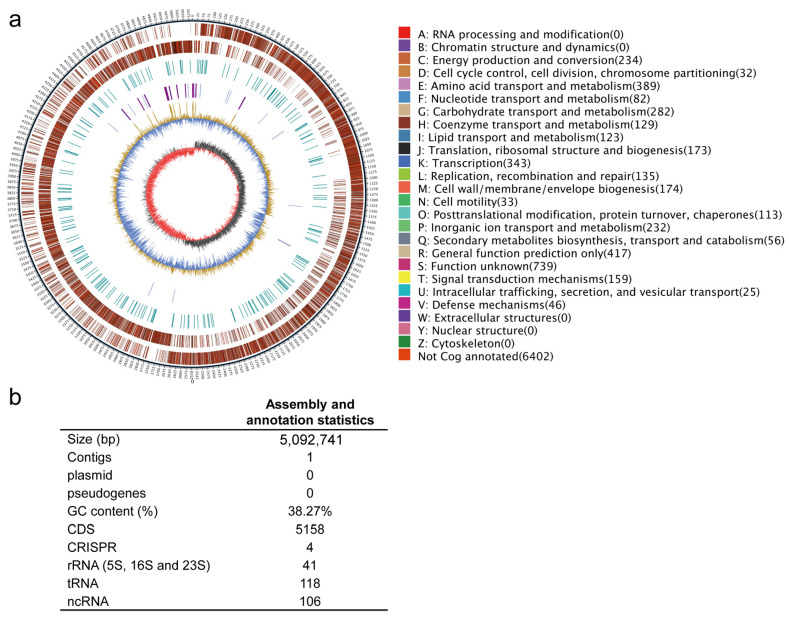
Results obtained from whole-genome analysis of *P. megaterium* ZS-3. (**a**) Circular map of the genome. The outermost circle is a marker for genome size; each scale bar is 5 kb. The second and third circles are the genes on the positive and negative strands of the genome, respectively, in which the different colours represent different COG functional classifications. The fourth circle shows the repeat sequences. The fifth circle shows tRNA and rRNA, in which blue indicates tRNA and purple indicates rRNA. The sixth circle shows the GC content; the light-yellow part indicates that the GC content of the region is higher than the average GC content of the genome, and the higher the peak is, the greater the difference from the average GC content is; the blue part indicates that the GC content in the region is lower than the average GC content in the genome. The innermost circle shows GC skew; dark grey represents regions with greater G content than C content, and red represents regions with greater C content than G content. (**b**) Genome assembly and annotation information.

Multigene phylogenetic analyses indicated that ZS-3 belongs to *P. megaterium* (Figure S1). The genome of ZS-3 is a circular chromosome with a full length of 5,092,741 bp and an average GC content of 38.27% (Figure 4a,b). The whole genome encodes 5158 protein-coding sequences, 118 tRNA genes, 41 rRNA genes, and 106 others noncoding RNAs. It contains 4 clustered regularly interspaced palindromic repeats sequences (CRISPR), 4 gene islands, 4 prophages, and 6 gene clusters. No plasmids or pseudogenes were detected in the genome of strain ZS-3 (Figure 4b).

### 2.5. Genome Mining for Stress Survival/Alleviation

The abundance of genetic elements involved in osmotic stress tolerance in the ZS-3 genome is consistent with functional data showing that ZS-3 is tolerant to moderate salt concentrations. We identified the presence of several key genes involved in osmotic stress tolerance, including sodium and chloride transporter proteins, K^+^ transport systems, compatible substance synthesis and transport systems (e.g., glycine betaine, alginate, pro, and glu), and membrane integrity and protective mechanisms (e.g., cardiolipin, lipophilin, and fatty acid desaturase) (Table S1). Furthermore, genome analysis revealed that heavy metal transporter/resistance genes were abundant in the ZS-3 genome (Table S2). These include several transporter/degradation genes (Zn, Co, Cu, Cd, Mn, Mg, arsenate, chromate, and fluoride), as well as the *arsC* gene, encoding arsenate reductase. The resistance of micro-organisms to heavy metals is essential for their survival in soil. Strains with multiple heavy metal resistance genes can survive in environments contaminated with heavy metals.

### 2.6. Genes Involved in Pgp

**Figure 5:**
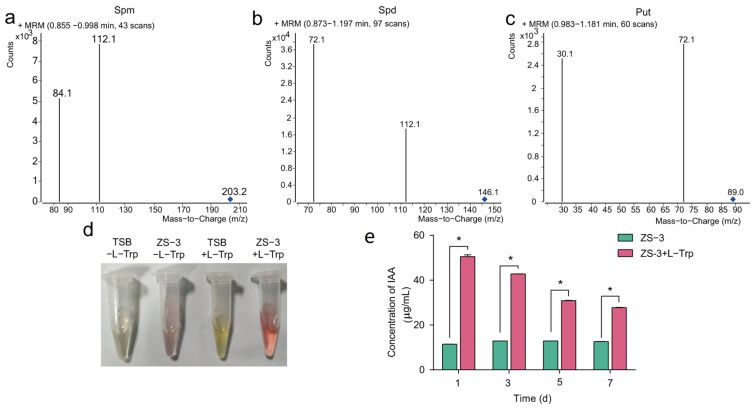
Polyamines and IAA secreted by *P. megaterium* ZS-3. Ion mass spectra of spm (**a**), spd (**b**), and put (**c**) targeted using HPLC-MS. (**d**) Color development reaction of Salkowski reagent mixed with cell supernatant. TSB-L-try: TSB medium without L-Try addition, ZS-3- L-try: ZS-3 cell supernatant without L-Try addition, TSB+ L-try: TSB with L-Try addition, ZS3+ L-try: ZS-3 cell supernatant with L-Try addition. (**e**) Effect of TSB medium with or without L-Try on IAA production by ZS-3 at 1 d, 3 d, 5 d, and 7 d. Values represent the means of three biological experiments, each in three technical replicates. Error bars represent SE. Asterisks on the bars indicate significant differences between treatments based on *t* test analysis (*p* < 0.05).

Polyamines such as putrescine (put), spermidine (spd), and spermine (spm) play important roles in plant growth promotion. Several polyamine metabolism and transporter genes were identified in the genome of strain ZS-3 (Table S3); these genes convert aa to plant-growth-promoting substances. In addition, we examined the cell supernatants of ZS-3 based on HPLC-MS and confirmed that strain ZS-3 could secrete put, spd, and spm (Figure 5a).

The production of indole-3-acetic acid (IAA) directly promotes the growth of plant roots. Whole-genome sequencing confirmed that strain ZS-3 possesses genes associated with IAA synthesis and tryptophan (trp) synthesis (Table S3), such as aldehyde dehydrogenase, suggesting that IAA secretion by strain ZS-3 is try-dependent. Consequently, we assessed the try-dependent production and secretion of IAA from strain ZS-3. Batch cultures of strain ZS-3 were grown in the presence or absence of try, and cell supernatants were analysed for IAA using the methods of Salkowski. The IAA yields of ZS-3 were 11.48, 12.87, 12.86, and 12.60 μg/mL at 1 d, 3 d, 5 d, and 7 d, respectively (Figure 5c). After L-tryptophan (L-Trp) was exogenously added, the cell supernatants of strain ZS-3 showed a darker pink color, indicating that the ability of strain ZS-3 to secrete IAA was significantly increased (Figure 5b). The quantitative results showed that the content of IAA secreted by strain ZS-3 increased 3.40-fold, 2.32-fold, 1.40-fold, and 1.20-fold at 1 d, 3 d, 5 d, and 7 d, respectively, after L-Trp was added (Figure 5c). In addition, γ-aminobutyric (GABA) biosynthesis, degradation, and transport genes were identified in the ZS-3 genome (Table S3).

Several volatile organic compounds (vocs) are produced by *Bacillus* strains and positively affect plant growth and defence responses. Genomic analysis revealed genes and pathways involved in the metabolism of acetyl urea and butanediol. In addition, DMS-, 1-3-propanediol-, and nitric oxide-related genes were identified in the genomic data (Table S4).

Genes involved in the metabolism of nitrogen, sulfur, and phosphorus were identified in the ZS-3 genome. ZS-3 has nitrate and nitrite reductase genes, as well as several genes involved in nitrate/nitrite transport (Table S5). In addition, urea degradation and transport genes were identified in the genome, as well as three ammonium transporter protein genes (Table S5). Sulfur metabolic pathways were also identified in the ZS-3 genome, including sulfate reduction and metabolism genes (Table S5). Previous studies have shown that ZS-3 can solubilise inorganic phosphorus through secreting organic acids, and genomic data confirmed the presence of phosphate transport system genes, as well as the alkaline phosphatase genes *phoA* and *phoD* (Table S5).

### 2.7. Biocontrol Activities

Genomic analysis revealed the presence of several genes involved in the production of antimicrobial compounds, such as the phosphate biosynthesis cluster and the gene encoding a type III polyketide synthase of the chalcone/stilbene synthase family (Table S6). In addition, the ZS-3 genome contains a cluster of siderophore biosynthesis genes and a cluster of genes that encode the siderophore transport system (Table S7), which contains homologues of the *yfhA*, *yfiZ,* and *yfiY* genes. The results from the chromium azurol S (CAS) assay indicated the presence of siderophores in the ZS-3 fermentation product (Figure S2). Plate confrontation tests have shown that ZS-3 is antagonistic to a variety of pathogens, including *Fusarium oxysporum*, *Botrytis cinerea*, *F*. *graminearum,* and *Botryosphaeria dothidea* (Figure S3).

### 2.8. Root Colonisation Study: GFP Marker Analysis

**Figure 6:**
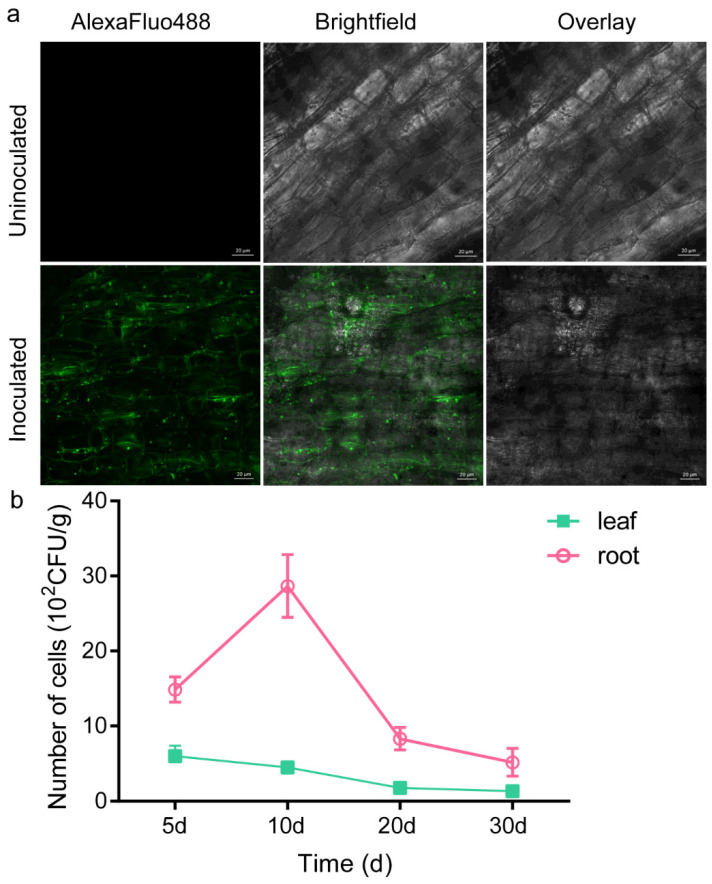
Quantification and visualisation of *P. megaterium* ZS-3-GFP in *C*. *camphora* roots and leaves. (**a**) Quantification of the ZS-3-GFP community in camphor roots and leaves by selection of medium and fluorescence microscopy at 5, 10, 20, and 30 d. (**b**) Colonisation by ZS-3-GFP in the roots was examined by confocal laser scanning microscopy at 30 d. Values represent the means of six biological experiments, each in three technical replicates. Error bars represent SE.

Core genes involved in the assembly of flagella, such as *flhAEFGHJLMNOPQRSSTW*, *flgBCDEGKL*, *fliACEFGHJ*, *motA,* and *motB*, were detected in the genome of ZS-3 (Table S8). The localisation of strain ZS-3 on the root surface of *Cinnamomum camphora* (L.) Presl was examined by colony counting and fluorescence microscopy. The population dynamics of the GFP-tagged ZS-3 strain (ZS-3-GFP) were determined via resolution of the GFP-tagged bacterial population from the roots and leaves at different time points. ZS-3-GFP was detected in the leaves and roots of seedlings (Figure 6a). The colony count in the leaf samples for ZS-3-GFP was 6 × 10^2^ cfu/g, while in the root samples, the colony count was 14.86 × 10^2^ cfu/g. The colony count for ZS-3-GFP in the leaves showed a decreasing trend and was 0.8 × 10^2^ cfu/g at 30 d (Figure 6a). However, the colony count in the roots increased at 10 d but then decreased to 5.2 × 10^2^ cfu/g at 30 d. The above results indicate that ZS-3 successfully colonised the roots and leaves of the plants. Confocal laser scanning microscopy further verified that ZS-3-GFP colonised on the root surface (Figure 6b).

### 2.9. Regulation of Soil Enzyme Activity by P. megaterium ZS-3

**Figure 7:**
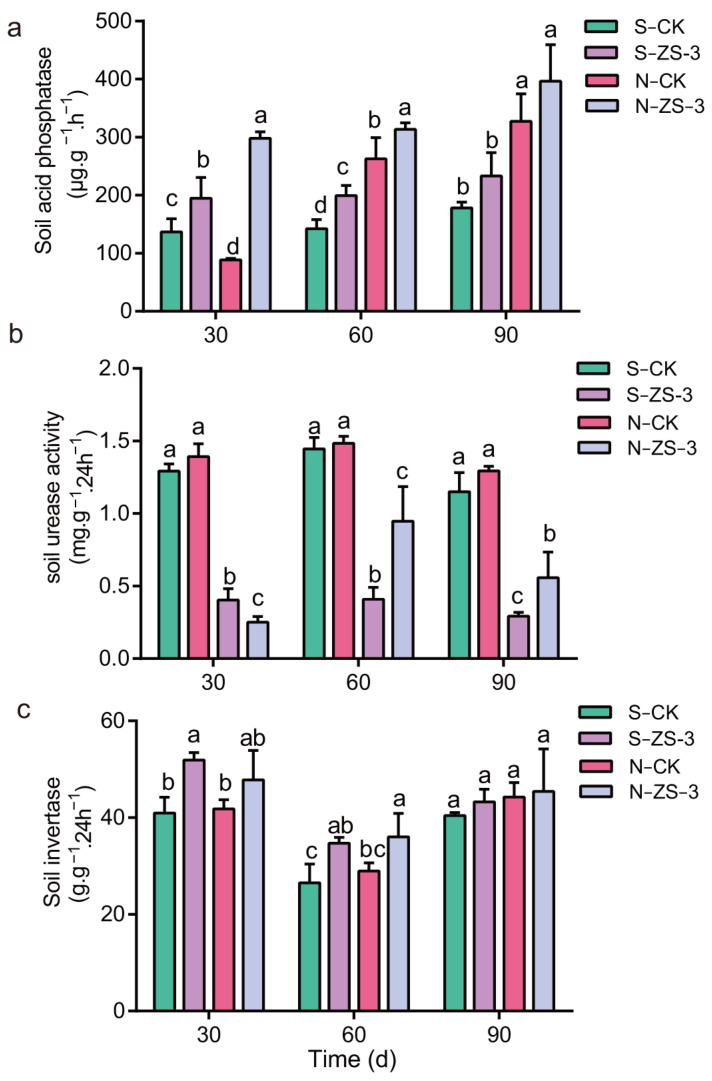
The application of *P. megaterium* ZS-3 improved ACP and urease activities in saline and neutral soils. Soil ACP (**a**), urease (**b**), and invertase (**c**) were assayed at 30 d, 60 d, and 90 d. S indicates saline soil (pH 8.15), and N indicates neutral soil (pH 6.8). CK represents treatments inoculated with sterile water, and ZS-3 represents treatments inoculated with ZS-3. Values represent the means of five biological experiments, each in three technical replicates. Error bars represent SE. Different lowercase letters above the bars represent significant differences based on one-way ANOVA (*p* < 0.05).

Under salt stress, strain ZS-3 significantly increased the biomass of *Arabidopsis thaliana*, and similar results were obtained with *C. camphora* seedlings. Soil enzyme activity was monitored in saline and neutral soils, and the results showed that ZS-3 was effective in increasing the biomass of the above and belowground parts of *C*. *camphora* in both soils (Tables S9 and S10). ZS-3 also effectively increases the activity of acid phosphatase (ACP), urease, and invertase in the soil. As shown in Figure 7a, the ACP activity of both soils showed an increasing trend after inoculation with strain ZS-3 as the cultivation time increased. The ACP activity increased by 17.76% (30 d), 9.86% (60 d), and 13.12% (90 d) in saline soil inoculated with ZS-3 (S-ZS-3) compared with saline soil inoculated with ddH_2_O (S-CK). ACP activity increased by 14.29% (30 d), 8.45% (60 d), and 10.49% (90 d) in neutral soil inoculated with ZS-3 (N-ZS-3) compared to that in soil inoculated with ddH_2_O (N-CK). In neutral soils, ACP activity increased by 14.29% (30 d), 8.45% (60 d), and 10.49% (90 d) in the N-ZS-3 treatment compared to the N-CK treatment. However, the effect of ZS-3 on soil urease activity was only monitored in neutral soils, and no significant effect on urease activity in saline soils was observed. As shown in Figure 7b, strain ZS-3 significantly improved urease activity in neutral soils at 60 d and 90 d. In addition, the application of ZS-3 significantly increased the activity of soil invertases. Soil urease activity increased by 26.69% and 14.35% in saline and neutral soils, respectively, compared to the control at 30 d. This difference in activity was observed at 60 d, but at 90 d, the promotion effect of ZS-3 on urease activity disappeared (Figure 7c). Therefore, the effect of ZS-3 on soil urease activity may be time-sensitive.

## 3. Discussion

The challenges faced by bacteria that inhabit soil include frequent fluctuations in osmotic stress [24]. *Bacillus* species must undergo many molecular and metabolic changes to survive hyperosmotic stress [25]. This study shows that strain ZS-3 is moderately salt-tolerant and its osmotic adaptation in response to different salt concentrations is integrated by several compatible substances. It is well-known that multiple salt stress resistance genes are present in individual bacteria, but the abundance of salt stress resistance genes in different species varies widely. The number of genes associated with choline, betaine uptake, and betaine biosynthesis ranged from 10 to 34 in the five halophilic *Pontibacillus* species [26]. The compatible solutes of halophilic bacteria are mainly polyols, polysaccharides, aas, and aa derivatives [27]. Ectoine and hydroxyectoine are the predominant osmotic solutes used for members of the gamma subclass of *Proteobacteria* and many gram-positive species [28]. Pro, Nδ-acetylornithine, and Nε-acetyllysine are found in low-G+C gram-positive bacteria, such as some *Bacillus* species and the related genera *Salinicoccus* and *Halobacillus* [28]. The abundance of genetic elements involved in osmotic stress tolerance in the ZS-3 genome coincides with the functional evidence that ZS-3 can tolerate salt stress. The results indicated that alginate and glu are compatible substances for strain ZS-3 in response to mild salt stress, while pro, K^+^, and EPS are involved in regulating the stress response of this strain at high salt stress levels.

Glu functions as the central metabolite that links carbon and nitrogen metabolism, as well as the main precursor for the de novo synthesis of pro [9]. This knowledge explains why the glu concentration in strain ZS-3 gradually decreased and the pro content increased as the NaCl concentration increased. Pro is a major compatible substance in some gram-positive bacteria [29]. However, pro concentrations in ZS-3 accumulated in large amounts only under high salt stress. *B. subtilis* is reported to physiologically prefer the uptake of betaine from the external environment to the ab initio synthesis of pro [9]. Genomic data show that ZS-3 contains *gbsB* and *betB*, which are involved in glycine betaine biosynthesis. Furthermore, ornithine-oxo-acid transaminase was localised in the pro synthesis pathway of ZS-3. This transaminase encodes a protein that catalyses the biosynthesis of pro- and polyamines via the ornithine pathway, potentially conferring resistance to drought and salt stress in plants [25].

The majority of bacterial genomes include five to nine different genes that encode monovalent cation/proton antiporters, which trade cytoplasmic Na^+^ and/or K^+^ for H^+^ from outside the cell [30]. Transmembrane proteins acting as Na^+^/H^+^ antiporters participate significantly in conserving intracellular pH, cellular sodium amount, homeostasis, and cell volume [30]. Five Na^+^/H^+^ antiporters are known in prokaryotes, including *NhaA*, *NhaB*, *NhaC*, *NhaD,* and *NapA* [31]. Strain ZS-3 contains a Na^+^/H^+^ antiporter (e.g., *NhaC*), multicomponent Na^+^/H^+^ antiporter subunit (*mnhA/B/C/D/E/F/G*), Ktr system potassium uptake protein (*ktrB/C/D*), and Ktr system potassium uptake protein (*ktrB/C/D*), which are genes involved in ion transport and osmoregulation. Adaptation to ionic hyperosmotic stress in *B. subtilis*, *B. cereus,* and *B. licheniformis* is a two-step process in which cells introduce K^+^ rapidly and transiently as an initial emergency response to rapid osmotic stress; then, cells replace these ions with compatible substances that do not interfere with cell physiology and biochemistry [25]. The concentration of K^+^ in ZS-3 accumulated substantially at salt concentrations greater than 5% rather than 3%. Therefore, the adverse physiological effects caused by 3% salt stress are tolerable for strain ZS-3. Strain ZS-3 only shows a stress response at salt concentrations above 5%; under this environment, strain ZS-3 integrates more cellular responses involved in the regulation of osmotic stress, such as the massive synthesis of EPS. Our results are similar to those from several studies in which salt stress was shown to enhance EPS production [32,33]. The release of EPSs into the surrounding environment is a strategy to retain water and avoid environmental desiccation caused by high salt stress [34,35]. In addition, bacteria reduce Na^+^ uptake and increase the water-holding capacity through EPS production to protect plants from abiotic stress [35].

There are four major trp-dependent IAA synthesis pathways in bacteria, including indole-3-pyruvate (IPyA), tryptamine (TAM), indole-3-acetonitrile (IAN), and indole-3-acetamide (IAM) [36]. *Burkholderia pyrrocinia* JK-SH007 contains all the genes needed for the IAM and TAM pathways but lacks key genes for the IPyA and IAN pathways [37]. Although there was no clear evidence that the IPyA or TAM pathways of strain ZS-3 were intact, the ability of strain ZS-3 to secrete IAA was significantly better than that of *B. pyrrocinia* JK-SH007 (Figure S4). Many bacteria can synthesise IAA through multiple incomplete pathways [38,39]. The strain ZS-3 genome contains five aldehyde dehydrogenases (EC:1.2.1.3), which convert indole-3-acetaldehyde to IAA. L-try is known as a major precursor important regulator of IAA biosynthesis [40]. Metagenome data showed that 75.0% (422) of rhizosphere bacteria could convert trp to intermediate metabolites in the IAA synthesis pathway, whereas 60.0% and 92.2% synthesised IAA from trp or intermediate metabolites, respectively [39]. In this study, L-try was added to the medium to analyse how L-try effects the yield of IAA synthesised by ZS-3, and the results were consistent with those previously described; L-try significantly enhanced the ability of rhizosphere bacteria to synthesise IAA [41,42]. More interestingly, L-try is among the main compounds in some plant exudates [43], which can help PGPRs (such as ZS-3) maximise the utilisation of bacterial IAA to improve crop growth and physiological functions.

Endogenous and exogenous polyamines can positively affect plant growth, yield, and stress resistance [44]. For example, spm can improve rice blast resistance through upregulating the jasmonic acid signalling pathway genes *PR1b* and *PBZ1* and the phytoalexin genes *CPS4* and *NOMT* [45]. Genomic data and HPLC-MS showed the ability of strain ZS-3 to synthesise put, spd, and spm. Polyamines are among the synthetic precursors of GABA [46]. Exogenous application of GABA induces disease resistance to *Penicillium expansum* by promoting the accumulation of endogenous GABA in apple fruit, which is regulated by enzyme activity and gene expression in polyamine anabolism and catabolism [47]. In addition, GABA has been shown to increase the ability of various plants to cope with various abiotic stresses, including low temperature [42], salt [48], PEG [49], and drought conditions [50]. There is an intact GABA synthesis, translocation, and degradation pathway in the genome of strain ZS-3, suggesting that GABA is a factor that facilitates the plant response to abiotic stress in ZS-3. However, further studies are needed to provide evidence to support this hypothesis.

The production of vocs by PGPR is an important mechanism for its contribution to plant growth; 2,3-Butanediol and acetoin produced by PGPR promote growth in *A. thaliana* [51]. Previous studies have shown that the vocs of ZS-3 promote the growth of plants, and the antagonistic effect of ZS-3 on fungi needs to be further explored. As biocontrol agents, PGPR produce extracellular secondary metabolites, such as surfactin, iturin, fengycin, 2,4-diacetylphloroglucinol, pyoluteorin, and phenazine; these metabolites inhibit the growth of bacterial and fungal pathogens and are attributed to the production of siderophores, vocs, cell-wall-degrading enzymes, extracellular chitinases, and protease activity [52,53]. *Bacillus amyloliquefaciens* MBI600 has been reported to produce the catecholate siderophore bacillibactin, which directly inhibits the growth of *Pseudomonas syringae* under iron-deficient conditions [54]. In addition, many PGPRs produced siderophores and significantly inhibited the growth of the pathogens *F. oxysporum* and *Rhizoctonia solani* [55]. In this study, the result indicated that strain ZS-3 could secrete siderophores under iron deficiency conditions, but the type of siderophores and antagonistic effect need further study.

Genomic and physiological studies have shown that various bacteria, such as *Pseudomonas*, *Rahnella*, *Burkholderia,* and *Bacillus,* utilise direct (phosphate solubilisation, nitrogen fixation, IAA synthesis, etc.) and indirect (antioxidant defence, volatile organic compounds, EPS, etc.) mechanisms to improve plant growth [25,56,57,58]. The present study investigated the potential direct mechanisms of action in the genome, including IAA synthesis, nitrogen, phosphorus and sulfur transport, metabolism, and degradation, as well as the synthesis of siderophores, polyamines, and GABA, which are beneficial for promoting plant growth. There are interspecies differences in the strategies used by micro-organisms to help plants deal with abiotic stress. For example, three strains of *Klebsiella* spp. showed significant differences in their strain activity despite containing the same PGP gene [59]. *B. pumilus* and *B. safensis* show more adaptations to plant-associated lifestyles than *P. megaterium* 16 PB based on an analysis of the genomic biosynthesis gene cluster and the amount of annotation of antifungal metabolites [60,61]. This result is consistent with the genomic genetic information of *P. megaterium* ZS-3, which has a significantly better potential for promoting plant growth than potential for biocontrol. The number of antimicrobial metabolite genes in the core genome is limited.

Genomic data revealed that strain ZS-3 contains one circular chromosome and zero plasmids. This species has a highly variable number of plasmids. The NCBI genome database shows that the number of plasmids in *P. megaterium* varies between 0 and 10 [13,16,62]. Five of these strains lacked natural plasmids [13], which is consistent with the ZS-3 genomic data. A typical feature of soil microbes is high numbers of rRNAs, which contribute to rapid growth, successful spore germination, and altered rapid response to nutrient availability [16]. Although ZS-3 was isolated from the leaf tissue of camphor, its rRNA count was much higher than that of the soil microbe *B. megaterium* NCT-2 [16]. The endophytic and rhizosphere colonisation ability of ZS-3-GFP was confirmed by isolating ZS-3-GFP from inoculated leaf and root tissues. In addition, strain ZS-3 promoted soil organic phosphorus decomposition and mineralisation, soil organic carbon conversion, and nitrogen metabolism by increasing soil ACP, sucrase, and urease activities [63,64]. Genes in the genome for nitrogen uptake, urea degradation, and translocation respond to the potential of strain ZS-3 to improve soil fertility in soils. The data obtained in this study provide additional clues to the potential application of *P*. *megaterium* ZS-3 in the development of eco-friendly biofertilisers that can contribute to the improvement of soil fertility and crop yield. The data also lay a foundation to further unravel plant–microbiome interactions and the mechanisms of microbial control of plant health.

## 4. Materials and Methods

### 4.1. Bacterial Cultures and Inoculation

LB medium was prepared with 10 g of tryptone, 5 g of yeast extract, and 10 g of NaCl (pH 7.4). Different salt concentration media were prepared based on LB medium. Briefly, LB medium was supplemented with 0.51, 0.86, 1.20, and 1.54 mol/L NaCl to obtain media with salt concentrations of 3%, 5%, 7%, and 9%, respectively. LB medium was used as a control. Solid LB medium was supplemented with 15 to 20 g of agar.

To prepare bacterium-only samples, ZS-3 was grown overnight in LB until it reached the exponential growth phase. The bacterial suspensions were inoculated into solid and liquid LB media and incubated at 28 °C. The cells of ZS-3 were collected by centrifugation and dried to constant weight, and the cell dry weight was determined. Then, 100 μL of the bacterial suspension (OD_600_ = 0.2) was inoculated into 10 mL of medium with different salt concentrations, mixed thoroughly, and added to a 96-well plate. The 96-well plates were placed in a Bioscreen C instrument (Oy Growthcurves, Turku, Finland) at 37 °C to determine the growth curve, and OD_600_ measurements were collected automatically every 2 h.

### 4.2. Extraction and Measurement of Intracellular Trehalose

A certain volume of precultures with different salt concentrations was collected and centrifuged at 8000 rpm for 15 min at 4 °C in an ice bath. The cell pellet was washed twice with isotonic NaCl solution, and the cells were collected again by centrifugation at 4 °C. After collection, the cells were treated with an ultrasonic cell analyser (Shunma Tech, Nanjing, China ) at low temperature. Centrifugation was performed at 4 °C and 6000 rpm for 15 min. The supernatant was retained and used to determine the content of trehalose at different salt concentrations with a trehalose content test kit (Comin Biotechnology, Suzhou, China).

### 4.3. Extraction and Measurement of the Intracellular Free aa Content

The overnight cultured bacteria were re-inoculated into 50 mL of LB different salinities and incubated at 28 °C for 48 h at 200 rpm. Subsequently, 50 mL of bacterial suspension was collected and placed in an ice bath and centrifuged at 8000 rpm for 10 min, and the supernatant was discarded. The precipitated bacterial bodies were rewashed twice with isotonic NaCl solution and centrifuged at 8000 rpm for 10 min at 4 °C to collect the cells. The cells were lysed with 5 mL of precooled 0.25 mol/L perchloric acid, mixed well, and left for 10 min. After centrifugation at 8000 rpm for 10 min at 4 °C, the supernatant was filtered through a membrane (0.22 µm, Millipore, MA, USA). The aa content was determined by an automatic amino acid analyser (Sykam, Munich, Germany). The volume flow rate of the buffer was 0.25 mL/min, the volume flow rate of ninhydrin (pH 5.2) was 0.45 mL/min, and the detection wavelengths were 570 nm and 440 nm.

### 4.4. Determination of the EPS Content

The overnight cultured bacteria were re-inoculated into 50 mL of LB different salinities and incubated at 28 °C for 72 h at 200 rpm for EPS quantification. Extraction procedures were performed according to a previously established method [65]. The extracted EPS was dialysed overnight at 4 °C against deionised water followed by vacuum drying and stored at 4 °C until further analysis. The concentration of EPS was determined by the phenol-sulfuric acid method [66] and the standard curve was prepared by using different concentrations of glucose.

### 4.5. Measurement of the Intracellular Na^+^ and K^+^ Ion Content

The overnight cultured bacteria were re-inoculated into 50 mL of LB and incubated at 28 °C for 48 h at 200 rpm. Subsequently, 50 mL of the bacterial suspension was collected. Centrifugation was performed at 8000 rpm for 10 min, and the supernatant was retained to measure Na^+^ and K^+^ concentrations in vitro. Precipitated bacterial cells were rewashed twice with PBS buffer (Sangon Biotech, Shanghai, China) and then centrifuged at 8000 rpm for 10 min. The collected cells were broken by an ultrasonic crusher (Shunma Tech, Nanjing, China) and centrifuged at 8000 rpm for 10 min. Then, the supernatant was used to determine the Na^+^ and K^+^ concentrations in vivo. LB medium without bacterial inoculum was used as a control. Na^+^ and K^+^ were extracted according to a previously established method [67]. The concentrations of Na^+^ and K^+^ were determined by flame photometry (AOE Instruments, Shanghai, China).

### 4.6. Determination of the Polyamine Content

Extraction of biogenic amines was carried out according to previously described methods [68]. Briefly, the sample supernatant was extracted three times with 5% trichloroacetic acid. After thorough mixing, the samples were centrifuged at 4 °C and 10,000× *g* for 3 min, and the supernatant was extracted and passed through an organic phase filter membrane (0.22 μm, Millipore, MA, USA). Characterisation of put, spd, and spm levels in the supernatant was performed using HPLC-MS (Agilent, CA, USA) as described previously [69]. The amine content was determined from external calibration curves drawn from standard solutions of put, spd, and spm.

### 4.7. Phytohormone Quantification and Visualisation by the Salkowski Method

Purified ZS-3 was grown in TSB medium with or without 5 mM L-Try (Yuanye Bio-Technology, Shanghai, China) and incubated in the dark at 37 °C. TSB medium with or without L-Trp addition was used as a negative control. IAA production and secretion were measured in culture supernatants using Salkowski reagent as described previously [70]. Briefly, the fermentation broth of strain ZS-3 reacted with Salkowski’s reagent and turned pink, indicating that strain ZS-3 produced IAA. After the pink color developed, the absorbance was measured at 530 nm and compared to a standard curve of IAA.

### 4.8. Root Colonisation Assay

The GFP gene sequence was amplified by PCR using pFB01 as a template, and the GFP primers were GFP-f (TCTCCGGAGCTCCCGGGATCCTTTGTAGGGCTCATCCATGCC) and GFP-v (CATGCGGGCCGGGTACCGGATCCGTTGTTGACTTTATCTACAAGGTGTGG). The pHIS1525-gfp plasmid was constructed by ligating the gfp gene into the corresponding restriction site (BamHI) of the pHIS1525 plasmid and then transformed into *Escherichia coli* DH5α. The gfp gene was ligated into the corresponding restriction site (*BamHI*) of the pHIS1525 Plasmid and then transformed into *Escherichia coli* DH5α. By screening positive transformants containing the gfp gene, the pHIS1525-gfp plasmid was obtained. The pHIS1525-gfp plasmid was transfected into ZS-3 cells according to a polyethylene glycol-mediated procedure [71]. The transformants ZS-3-GFP that emitted green fluorescence were screened using selective medium (LB medium containing 10 μg mL^−1^ tetracycline) and fluorescence microscopy (Carl Zeiss AG, Oberkochen, Germany) [72]. All constructed plasmids and transformants were validated by DNA sequencing (Springen Biotechnology, Nanjing, China).

ZS-3-GFP was inoculated into LB medium containing tetracycline (10 µg/mL) and incubated at 200 rpm for 24 h at 37 °C in a shaker. ZS-3-GFP cultures were centrifuged at 8000 rpm for 10 min, and then the cell pellet was suspended in sterile distilled water at a final concentration of 1 × 10^8^ cfu mL^−1^. To observe the dynamics of colonisation, annual *C. camphora* were used as sample, and each pot was inoculated with 100 mL of ZS-3-GFP bacterial suspension (1 × 10^8^ cfu mL^−1^). An equal volume of sterile water was used as the control. *C. camphora* were grown in nursery pots containing sterile soil (pH 6.8) with one seedling per pot. Each treatment had five biological replicates and three technical replicates. On the 5th, 10th, 20th, and 30th days post inoculation, fresh leaves and roots were collected. ZS-3-GFP strains were isolated from fresh leaves and roots on LB plates containing tetracycline. A fluorescence microscope (Carl Zeiss AG, Oberkochen, Germany) was used to determine the number of colonies of ZS-3-GFP. Images of the roots were captured under a confocal microscope (Carl Zeiss AG, Oberkochen, Germany) equipped with a 20× objective and an emission wavelength of 488 nm, and fluorescence was measured in the 500–550 nm range.

### 4.9. Estimation of Soil Enzyme Activity

Two-year-old *C. camphora* seedlings were transplanted into pots containing saline soil (pH 8.15) and neutral soil (pH 6.8), and one seedling was planted in each pot. Each pot was inoculated with 100 mL of a cell suspension of the ZS-3 strain (1 × 10^8^ cfu mL^−1^), and the treatment inoculated with an equal amount of sterile water served as a negative control. The following treatment groups were used: S-CK, S-ZS-3, N-CK, and N-ZS. Fifteen replicates were set up for each treatment. All other management practices were the same for each group during potting, and soil ACP, urease, and invertase activities were measured at 30 d, 60 d, and 90 d. Soil ACP, urease, and invertase activities under different treatments were measured by ACP activity test kits, soil urease test kits, and soil sucrase activity test kits (Comin Biotechnology, Suzhou, China).

### 4.10. Genome Sequencing and Bioinformatic Analysis

The ZS-3 genome was sequenced by the third-generation (Pac-Bio SMRT) sequencing technology method. Hifiasm was used for assembly, Circlator v1.5.5 was used for circularisation and adjustment of start sites, and Pilon v1.22 was used for further error correction using second-generation sequencing data, resulting in a final high-precision genome for subsequent analysis.

Prodigal v2.6.3 was used for gene prediction in the genome. RepeatMasker v4.0.5 was used to search the genome for repetitive sequences by comparing it with a database of known repetitive sequences. Infernal v1.1.3 was used to accurately predict the three classes of rRNA in the genome based on a covariance model. tRNAscan-SE v2.0 was used to search for tRNAs in the genome with high accuracy based on the sequence and secondary structure features of tRNAs and the corresponding promoter features. Infernal v1.1.3 predicts the presence of other ncRNAs in the genome based on covariance models. CRT v1.2 software was used to predict CRISPR.

Predicted gene sequences were compared with the nonredundant (Nr), gene ontology (GO), Kyoto Encyclopedia of Genes and Genomes (KEGG), direct homology cluster (eggNOG), protein family (Pfam), Swiss-Prot, TrEMBL, and other functional databases by BLAST and gene function annotation. The Draft Genome Shotgun sequence project of ZS-3 has been deposited under the CP129891 accession number at GenBank. *P. megaterium* ZS-3 is stored as No: M20231406 in the China Centre for Type Culture Collection (CCTCC).

### 4.11. Data Analyses

Statistical analysis of the data was performed using SPSS (IBM, Armonk, NY, USA). One-way analysis of variance (ANOVA), Student’s *t* test (*t* test), and Duncan’s multiple range test were performed. The data represented in the graphs are means ± standard deviations (SDs) of at least three replicate biological samples (*n* ≥ 3).

## Data Availability

The raw data supporting the conclusions of this article will be made available by the authors upon request.

## Supplementary Materials

The following supporting information can be downloaded at: https://www.mdpi.com/article/10.3390/ijms242115751/s1.
