# Supplementary material for: Mechanism of Salt Tolerance and Plant Growth Promotion in Priestia megaterium ZS-3 Revealed by Cellular Metabolism and Whole-Genome Studies
Source: Int J Mol Sci. 2023 Oct 30;24(21):15751. doi: 10.3390/ijms242115751 (PMC10647267; doi:10.3390/ijms242115751)
Supplement: Supplementary file 1 [file ijms-24-15751-s001.zip › ijms-2605145-supplementary.pdf]

Supplementary Material

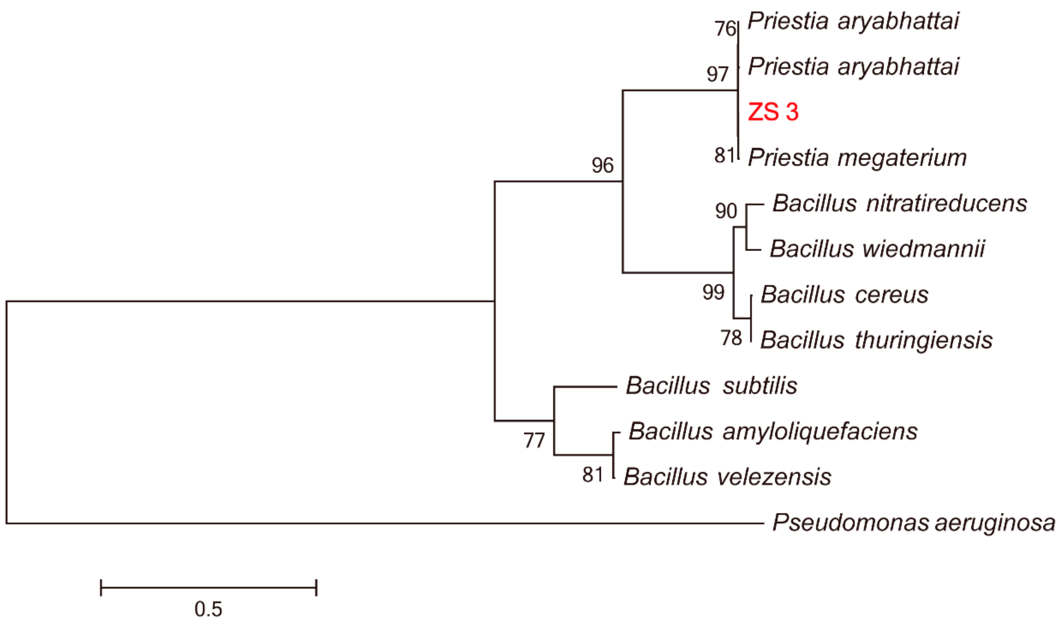

Figure S1 Maximum likelihood (ML) phylogenetic tree generated from analysis of a combined 16s and gyrB sequences dataset. *Pseudomonas aeruginosa* is the outgroup taxa.

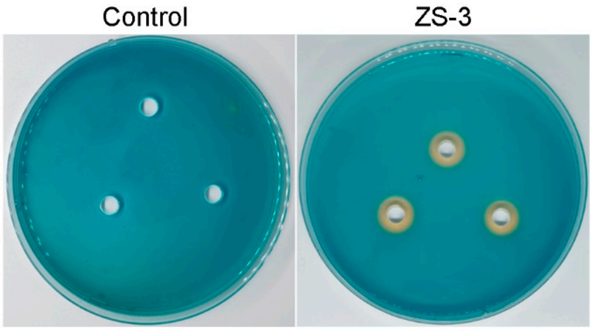

Figure S2 The ability of *P. megaterium* ZS-3 to secrete siderophores was detected in CAS medium.

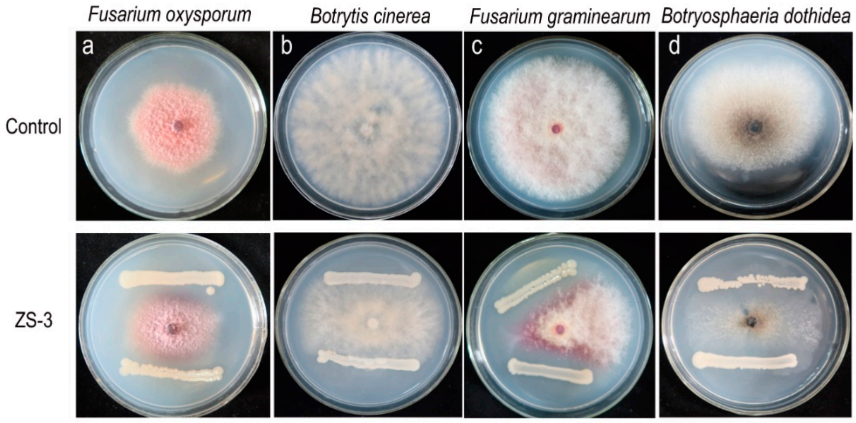

Figure S3 Effect of antagonistic cultures of *P. megaterium* ZS-3 with the following

pathogens: a. *F. oxysporum*; b. *B. cinerea*; c. *F. graminearum*; d. *Botryosphaeria dothidea*. The control group was a single inoculated treatment with the pathogen and the others were antagonistic cultures of ZS-3 with the pathogen.

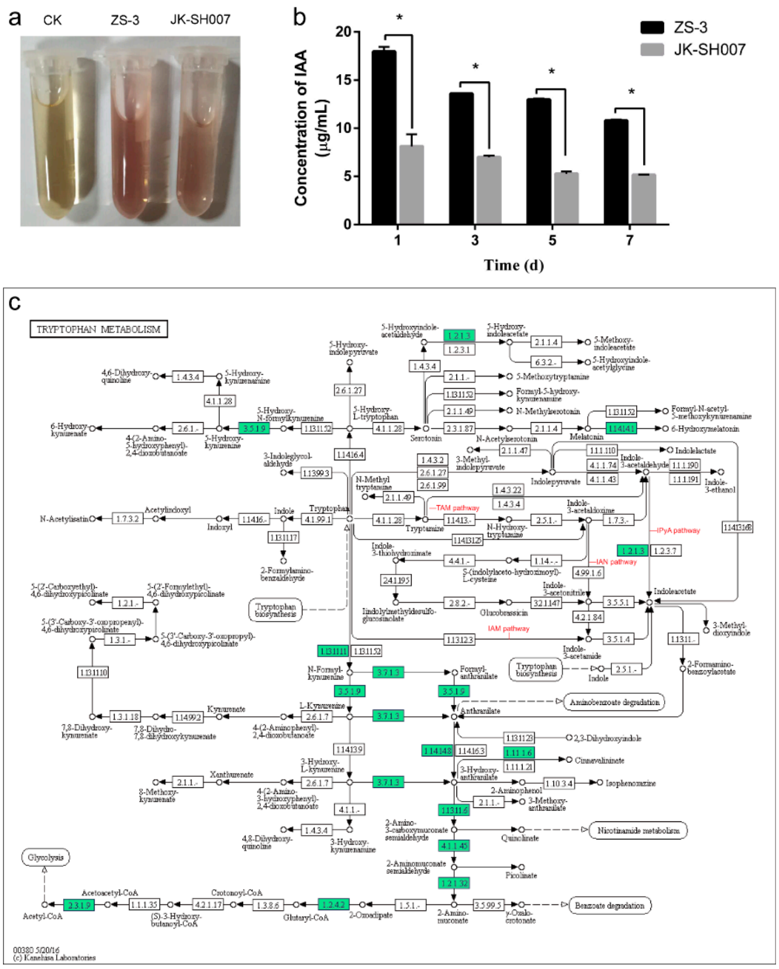

Figure S4 Analysis of the IAA secretion and KEGG synthesis pathways. Colour development reaction (a) and quantitative analysis (b) of Salkowski's reagent mixed with ZS-3 and JK-SH007 cell supernatants. CK is TSB medium without bacteria inoculation. Putative "KEGG pathway" of trp metabolism and IAA biosynthetic pathways in the *P. megaterium* ZS-3. The numbers in the boxes represent the EC numbers of key enzymes in the metabolic pathway, and the green boxes indicate the presence of genes corresponding to the relevant enzymes in the genome. Values represent the means of three biological experiments, each in three technical replicates. Error bars represent SE. Asterisks on the bars indicate significant differences between the treatments analysed according to the t test ($P < 0.05$).

Table S1- Genes involved in osmotic stress tolerance.

Gene ID	Gene	Annotation	EC numbers	Pathway
**GE004605**	*rocD-*	Ornithine--oxo-acid transaminase	EC:2.6.1.13	**Arginine and proline metabolism**
**GE000066<br>GE002967**	*proA*	Glutamate-5-semialdehyde dehydrogenase	EC：1.2.1.41	**Proline biosynthesis**
**GE000065<br>GE002968**	*proB*	Glutamate 5-kinase	EC：2.7.2.11	
**GE002969<br>GE003249<br>GE000862**	*proC*	Pyrroline-5-carboxylate reductase	EC:1.5.1.2	
**GE000222<br>GE000297<br>GE000298<br>GE001454**	*proDH*	Proline dehydrogenase	EC：1.5.5.2	
**GE003159**	*gltB*	Glutamate synthase [NADPH] small chain	EC:1.4.1.13	**Glutamate biosynthesis**
**GE003158**	*gltD*	Glutamate synthase [NADPH] large chain	EC：1.4.1.13	
**GE001177**	*glnA*	Glutamine synthetase	EC:6.3.1.2	**Glutamine biosynthesis**
**GE003736**	*opuAC*	Glycine betaine/proline transport system substrate-binding protein	--	**Glycine betaine/proline transport and biosynthesis**
**GE003737**	*proW*	Glycine betaine/proline transport system permease protein	--	
**GE003738**	*opuAA*	Glycine betaine/proline transport system ATP-binding protein	EC:7.6.2.9	
**GE003739**		Glycine betaine catabolism A	--	
**GE003927<br>GE003928**	*opuD*	Glycine betaine transporter OpuD	--	
**GE000200<br>GE002996<br>GE004398**	*betB*	Betaine-aldehyde dehydrogenase	EC:1.2.1.8	
**GE000199**	*gbsB*	Choline dehydrogenase	EC:1.1.1.1	
**GE001261**	*ptbA*	PTS glucose transporter subunit IIA	EC:2.7.1.-	**Trehalose biosynthesis**
**GE004306**	*treC*	Trehalose-6-phosphate hydrolase	EC:3.2.1.93	
**GE001851<br>GE001950<br>GE002623<br>GE004211<br>GE004788**	*gltP*	Proton glutamate symport protein	--	**Glutamate transport**
**GE003524<br>GE004532**		Na(+)/H(+) antiporter NhaS5		**Sodium and chloride transporters**
**GE004011**	*NhaC*	Na(+)/H(+) antiporter NhaC		
**GE004063**	*natB*	Sodium transport system permease protein	--	
**GE004064**	*natA*	Sodium transport system ATP-binding protein	EC:7.2.2.4	
**GE000370**	*mnhA*	Multicomponent Na+:H+ antiporter subunit A	--	
**GE000369**	*mnhB*	Multicomponent Na+:H+ antiporter subunit B	--	
**GE000368**	*mnhC*	Multicomponent Na+:H+ antiporter subunit C	--	
**GE000367**	*mnhD*	Multicomponent Na+:H+ antiporter subunit D	--	
**GE000366**	*mnhE*	Multicomponent Na+:H+ antiporter subunit E	--	
**GE000365**	*mnhF*	Multicomponent Na+:H+ antiporter subunit F	--	
**GE000364**	*mnhG*	Multicomponent Na+:H+ antiporter subunit G	--	
**GE005122**	*yfbS*	Ca-activated chloride channel homolog	--	
**GE002415**	*nhaK*	Sodium, potassium, lithium and rubidium/H($^+$) antiporter	--	
**GE001374<br>GE003450**	*ktrB*	Ktr system potassium uptake protein B	--	**Low-affinity potassium transport system**
**GE001373<br>GE003940**	*ktrC*	Ktr system potassium uptake protein C	--	
**GE004044**	*ktrD*	Ktr system potassium uptake protein D	--	
**GE000117<br>GE003785<br>GE004546<br>GE004761**	*clsAB*	Cardiolipin synthase A/B	EC:2.7.8.-	**Cardiolipin biosynthesis**
**GE001334<br>GE003236<br>GE004417**	*ltaS*	Lipoteichoic acid synthase	EC:2.7.8.20	**Lipoteichoic acid biosynthesis**

**GE003788**	*des*	Fatty acid desaturase	--	**Fatty acid membrane modifications**

Table S2- Genes involved in heavy metal transport and resistance.

Gene ID	Gene	Annotation	EC numbers	Pathway
**GE000408**	*znuA*	Zinc transport system substrate-binding protein	--	**Zinc transport**
**GE000750**		Zinc transport system ATP-binding protein	EC:7.2.2.-	
**GE000751**		Zinc transport system permease protein	--	
**GE003468**<br>**GE003705**<br>**GE004018**		Zinc transporter, ZIP family	--	
**GE004866**	*zitB*	Cobalt-zinc-cadmium efflux system protein	--	**Cobalt-zinc-cadmium transport**
**GE001208**<br>**GE004500**	*cutC*	Copper homeostasis protein cutC	--	**Copper resistance transport**
**GE002267**	*copD*	Copper resistance protein D	--	
**GE002266**	*copC*	Copper resistance protein C	--	
**GE003352**	--	Copper-exporting P-type ATPase	--	
**GE003353**	*copZ*	Copper chaperone CopZ	--	
**GE003342**<br>**GE003529**<br>**GE004204**	*arsB*	Arsenical pump membrane protein	--	**Arsenite transport and reduction**
**GE000303**	*arsC*	Arsenate reductase (glutaredoxin)	EC:1.20.4.1	
**GE003530**		Arsenate reductase (thioredoxin)	EC:1.20.4.4	
**GE002245**<br>**GE002246**	*chrA*	Chromate transporter	--	**Chromate reduction and transport**
**GE002110**<br>**GE004869**	*cadA*	Cadmium-transporting ATPase	--	**Cadmium transport**
**GE002111**	*cadC*	Cadmium resistance transcriptional regulatory protein CadC	--	
**GE004866**	*czcD*	Cadmium, cobalt and zinc/H(+)-K(+) antiporter	--	
**GE002726**	*mntH*	Manganese transport protein	--	**Manganese ransport**
**GE000140**	*mntP*	Putative manganese efflux pump MntP	--	
**GE004533**	*mgtE*	Magnesium transporter MgtE	--	**Magnesium trasnport**
**GE002360**<br>**GE003322**<br>**GE004110**<br>**GE004533**<br>**GE004906**	*corA*	Magnesium transporter	--	
**GE000384**<br>**GE001320**	*mgtC*	Putative Mg^{2+} transporter-C (MgtC) family protein	--	
**GE004699**<br>**GE004700**	*crcB*	Fluoride exporter	--	**Fluoride transport**

Table S3- Genes involved in phytohormone production/modulation.

Gene ID	Gene	Annotation	EC numbers	Pathway
**GE003925**	*speA*	Arginine decarboxylase	--	**Putrescine biosynthesis**
**GE000113 GE002997**	*speB*	Agmatinase	EC:3.5.3.11	
**GE000524**	*speH*	S-adenosylmethionine decarboxylase	EC:4.1.1.50	**Spermidine biosynthesis**
**GE000112 GE004482**	*speE*	Spermidine synthase	EC:2.5.1.16	
**GE004397**	*pat*	Putrescine aminotransferase	EC:2.6.1.82	**Putrescine degradation**
**GE003640**	*speG*	Spermidine/Spermine N (1)-acetyltransferase		**Acetylspermidine formation**
**GE005012**	*potD*	Spermidine/putrescine transport system substrate-binding protein	--	**Polyamine transport**
**GE005013**	*potC*	Spermidine/putrescine transport system permease protein	--	
**GE005014**	*potB*	Spermidine/putrescine transport system permease protein	--	
**GE005015**	*potA*	Spermidine/putrescine transport system ATP-binding protein	EC:7.6.2.11	
**GE004395 GE004784**	*puuP*	Putrescine importer	--	
**GE000956**	*trpB*	Tryptophan synthase beta chain	EC:4.2.1.20	**Tryptophan biosynthetic**
**GE000957**	*trpA*	Tryptophan synthase alpha chain	EC:4.2.1.20	
**GE001688**	*kynA*	Tryptophan 2,3-dioxygenase	EC:1.13.11.11	**Tryptophan metabolism**
**GE001883 GE002878 GE003111 GE003378 GE003717**	-	Aldehyde dehydrogenase (NAD+)	EC:1.2.1.3	**IAA biosynthesis**
**GE002787**	*gadB*	Glutamate decarboxylase	EC:4.1.1.15	**GABA biosynthesis and transport**
**GE001201 GE003827**	*gabD*	Succinate-semialdehyde dehydrogenase	EC:1.2.1.16 1.2.1.79 1.2.1.20	
**GE002879 GE004199 GE004319**	*gabT*	4-aminobutyrate aminotransferase	EC:2.6.1.19	
**GE001456 GE004499**	*gabP*	GABA permease	--	

Table S4- Genes involved in vocproduction/degradation.

Gene ID	Gene	Annotation	EC numbers	Pathway
**GE004474**	*aldC*	Alpha-acetolactate decarboxylase		**Acetoin biosynthesis**
**GE000582 GE000583 GE003566 GE004310 GE004473**	*alsS*	Acetolactate synthase		
**GE000469**	*acuA*	Acetoin utilization protein AcuC	--	**Acetoin catabolism**
**GE000470**	*acuB*	Acetoin utilization protein AcuB	--	
**GE000471**	*acuC*	Acetoin utilization protein AcuA	EC:2.3.1.-	
**GE003389**	*acoA*	Acetoin:2,6-dichlorophenolindophenol oxidoreductase subunit alpha	--	
**GE003388**	*acoB*	Acetoin:2,6-dichlorophenolindophenol oxidoreductase subunit beta	--	
**GE003387**	*acoC*	Dihydrolipoyllysine-residue acetyltransferase component of acetoin cleaving system	--	
**GE000835 GE003386 GE003930**	*acoL*	Dihydrolipoyl dehydrogenase	EC:1.8.1.4	
**GE003385**	*acoR*	Acetoin dehydrogenase operon transcriptional activator AcoR	--	
**GE003384**	*bdhA*	(R,R)-butanediol dehydrogenase	EC:1.1.1.4 1.1.1.- 1.1.1.303	**Butanediol degradation**
**GE001552 GE002594**	*dhaT*	1,3-propanediol dehydrogenase	--	**1,3-propanediol catabolism**
**GE003682 GE004390**	*dmoA*	Dimethyl-sulfide monooxygenase	--	**Dimethyl sulfone degradation**
**GE001930**	*sfnG*	FMNH(2)-dependent dimethylsulfone monooxygenase	--	

Table S5 - Genes involved in nitrogen, sulfur, and phosphorus metabolism.

Gene ID	Gene	Annotation	EC numbers	Pathway
**GE000658**	*pstS*	Phosphate transport system substrate-binding protein	--	**Phosphate transport**
**GE000659**	*pstC*	Phosphate transport system permease protein	--	
**GE000660**	*pstA*	Phosphate transport system permease protein	--	
**GE000763**	*pstB*	Phosphate transport system ATP-binding protein	EC:7.3.2.1	
**GE003766 GE004055**	*phoA*	Alkaline phosphatase	EC:3.1.3.1	**Phosphate assimilation**
**GE000211**	*phoD*	Alkaline phosphatase D	EC:3.1.3.1	
**GE000057**	*phoR*	Alkaline phosphatase synthesis sensor protein	--	
**GE000517**	*phoP*	Alkaline phosphatase synthesis transcriptional regulatory protein	--	
**GE002287**	*ureC*	Urease subunit alpha	EC:3.5.1.5	**Urea degradation and transport**
**GE002286**	*ureB*	Urease subunit beta	EC:3.5.1.5	
**GE002285**	*ureA*	Urease subunit gamma	EC:3.5.1.5	
**GE002293**	*ureH*	Urease accessory protein UreH	--	
**GE002291**	*ureD*	Urease accessory protein UreD	--	
**GE002290**	*ureG*	Urease accessory protein UreG	--	
**GE002289**	*ureF*	Urease accessory protein UreF	--	
**GE002288**	*ureE*	Urease accessory protein UreE	--	
**GE000380**	--	Adenylyl-sulfate kinase		**Sulfur metabolic and transport**
**GE000377**	*cysH*	Phosphoadenylyl-sulfate reductase	EC:1.8.4.8 1.8.4.10	
**GE000378**	*cysP*	Sulfate permease		
**GE000379**	*sat*	Sulfate adenylyltransferase	EC:2.7.7.4	
**GE000380**		Adenylylsulfate kinase	EC:2.7.1.25	
**GE000566**		Sulfonate transport system ATP-binding protein	EC:3.6.3.-	
**GE000567**		Sulfonate transport system substrate-binding protein		
**GE000568**		Sulfonate transport system permease protein		
**GE002152**	*cysJ*	Sulfite reductase (NADPH) flavoprotein alpha-component	EC:1.8.1.2	
**GE002153**	*cysI*	Sulfite reductase (NADPH) hemoprotein beta-component	EC:1.8.1.2	
**GE003131**	*cysA*	Sulfate/thiosulfate import ATP-binding protein CysA	EC:7.3.2.3	
**GE003132**	*cysW*	Sulfate transport system permease protein CysW	--	
**GE003133**	*cysT*	Sulfate transport system permease protein CysT	--	
**GE003134**	--	Sulfate/thiosulfate transport system substrate-binding protein	--	
**GE003291**	--	Thiosulfate/3-mercaptopyruvate sulfurtransferase	EC:2.8.1.1 2.8.1.2	
**GE000570 GE004388**	--	Alkanesulfonate monooxygenase	EC:1.14.14.5	
**GE000569**	--	FMN reductase (NADPH)	EC:1.14.14.5	
**GE004123 GE004485**	*nirB*	Nitrite reductase (NADH) large subunit	EC:1.7.1.15	**Dissimilatory nitrate reduction**
**GE004122**	*nirD*	Nitrite reductase (NADH) small subunit	EC:1.7.1.15	
**GE004484**	*nasA*	Assimilatory nitrate reductase catalytic subunit	EC:1.7.99.-	
**GE004483**	--	MFS transporter, NNP family, nitrate/nitrite transporter	--	**Nitrate/nitrite transporter**
**GE004120**	*nirC*	Nitrite transporter	--	
**GE002618 GE002330 GE004609**	*amt*	Ammonium transporter, Amt family		**Ammonia transport**

Table S6- Genes involved in the production of antagonistic traits.

Gene ID	Gene	Annotation	EC numbers	Pathway
**GE004479**<br>**GE005004**	*murF*	UDP-N-acetylmuramoyl-tripeptide--D-alanyl-D-alanine ligase	EC:6.3.2.10	**Phosphonate biosynthesis**
**GE004478**	*aepX*	Phosphoenolpyruvate phosphomutase	--	
**GE004477**	*aepY*	Phosphonopyruvate decarboxylase	--	
**GE002042**	*phnX*	Phosphonoacetaldehyde hydrolase	--	
**GE002043**	*phnW*	2-aminoethylphosphonate--pyruvate transaminase	--	
**GE002841**	-	MBL fold metallo-hydrolase	--	**Phosphonate and phosphinate metabolism**
**GE001239**<br>**GE001300**<br>**GE001310**	-	N-acetyltransferase	--	
**GE002709**	-	L-amino acid N-acyltransferase MnaT	--	
**GE001893**	-	LysR family transcriptional regulator	--	**Alkylresorcinol, alkylpyrones biosynthesis**
**GE001201**	-	Glutarate-semialdehyde dehydrogenase	--	
**GE003827**		Succinate-semialdehyde dehydrogenase [NADP(+)]	--	
**GE003825**	-	Type III polyketide synthase，Putative chalcone synthase	--	
**GE003824**	-	Isoprenylcysteine carboxyl methyltransferase	--	
**GE000624**	-	GTPase	--	

Table S7- Genes involved in iron transport and siderophore production.

Gene ID	Gene	Annotation	EC numbers	Pathway
**GE002693**	*fbpB*	Iron(III) transport system permease protein	--	**Iron(III) transport**
**GE001268**	*fbpC*	Fe(3+) ions import ATP-binding protein FbpC	--	
**GE004363**	*feoA*	Ferrous iron transport protein A	--	**Iron(II) transport**
**GE001714**<br>**GE004364**<br>**GE001713**	*feoB*	Ferrous iron transport protein B	--	
**GE000291**<br>**GE002064**<br>**GE002802**<br>**GE003268**<br>**GE003753**<br>**GE004386**	*fhuD*	Iron complex transport system substrate-binding protein	--	**Iron complex transport**
**GE000292**<br>**GE000293**<br>**GE002061**<br>**GE002062**<br>**GE002313**<br>**GE003754**<br>**GE003755**<br>**GE004384**<br>**GE004385**	*fhuB*	Iron complex transport system permease protein	--	
**GE000294**<br>**GE002063**<br>**GE002314**<br>**GE003078**	*fhuC*	Iron complex transport system ATP-binding protein	EC:7.2.2.-	
**GE001213**<br>**GE004878**	*rhbD*	Lysine 6-monooxygenase	--	
**GE001211**	*rhbC*	Siderophore biosynthesis protein RhbC	--	
**GE001213**<br>**GE004878**	*rhbE*	Siderophore biosynthesis protein RhbE	--	
**GE001215**	*rhbF*	Siderophore biosynthesis protein RhbF	--	
**GE000292**	*yfiZ*	Probable siderophore transport system permease protein YfhZ	--	**Siderophore transport**
**GE000293**	*yfhA*	Probable siderophore transport system permease protein YfhA	--	
**GE000291**	*yfiY*	Probable siderophore-binding lipoprotein YfiY	--	

Table S8- Genes involved in chemotaxis and motility.

Gene ID	Gene	Annotation	EC numbers	Pathway
**GE001106 GE003735**	*CheA*	Two-component system, chemotaxis family, sensor kinase CheA	**EC:2.7.13.3**	**Chemotaxis**
**GE001107**	*CheW*	Purine-binding chemotaxis protein CheW	--	
**GE001108**	*CheD*	Chemotaxis protein CheD	**EC:3.5.1.44**	
**GE000949 GE003734**	*CheR*	Chemotaxis protein methyltransferase CheR	**EC:2.1.1.80**	
**GE001105 GE002970**	*CheB*	Two-component system, chemotaxis family, CheB/CheR fusion protein	**EC:2.1.1.80 3.1.1.61**	
**GE001096**	*CheY*	Two-component system, chemotaxis family, chemotaxis protein CheY	**--**	
**GE003963**	*CheV*	Two-component system, chemotaxis family, chemotaxis protein CheV	**--**	
**GE000467 GE003218**	*MotA*	Chemotaxis protein MotA	**--**	
**GE000468 GE003217**	*MotB*	Chemotaxis protein MotB	**--**	
**GE002775 GE004721**	*Mcp*	Methyl-accepting chemotaxis protein	**EC:4.1.1.15**	
**GE001385**	*HemAT*	Heme-based aerotactic transducer	**-**	
**GE001102**	*FlhA*	Flagellar biosynthesis protein FlhA	--	**Flagellar assembly**
**GE001109**	*FliA*	RNA polymerase sigma factor for flagellar operon FliA	--	
**GE001080**	*FlgB*	Flagellar basal-body rod protein FlgB	--	
**GE001101**	*FlhB*	Flagellar biosynthetic protein FlhB	--	
**GE001081**	*FlgC*	Flagellar basal-body rod protein FlgC	--	
**GE004169**	*FliC*	Flagellin		
**GE001090**	*FlgD*	Flagellar basal-body rod modification protein FlgD	--	
**GE001092**	*FlbD*	Flagellar protein FlbD；Swarming motility protein SwrD	--	
**GE001082**	*FliE*	Flagellar hook-basal body complex protein FliE	--	
**GE001091**	*FlgE*	Flagellar hook protein FlgE	--	
**GE001083**	*FliF*	Flagellar M-ring protein FliF	--	
**GE001103**	*FlhF*	Flagellar biosynthesis protein FlhF		
**GE000165 GE000166**	*FlgG*	Flagellar basal-body rod protein FlgG	--	
**GE000186**	*FliS*	Flagellar protein FliS		
**GE001084**	*FliG*	Flagellar motor switch protein FliG		
**GE001104**	*FlhG*	Flagellar biosynthesis protein FlhG		
**GE001085**	*FliH*	Flagellar assembly protein FliH		
**GE001087**	*FliJ*	Flagellar FliJ protein		
**GE000194**	*FlgK*	Flagellar hook-associated protein 1 FlgK		
**GE000195**	*FlgL*	Flagellar hook-associated protein 3 FlgL		
**GE001093**	*FliL*	Flagellar FliL protein		
**GE001094**	*FliM*	Flagellar motor switch protein FliM		
**GE001095**	*FliN/FliY*	Flagellar motor switch protein FliN/FliY		
**GE001097**	*FliO/FliZ*	Flagellar protein FliO/FliZ		
**GE001098**	*FliP*	Flagellar biosynthetic protein FliP		
**GE001099**	*FliQ*	Flagellar biosynthetic protein FliQ		
**GE001100**	*FliR*	Flagellar biosynthetic protein FliR		
**GE000205**	*FliS*	Flagellar protein FliS		
**GE000206**	*FliT*	Flagellar protein FliT		
**GE000196**	*FliW*	Flagellar assembly factor FliW		
**GE000204**	*FliD*	Flagellar hook-associated protein 2		
**GE001086**	*FliS*	Flagellum-specific ATP synthase	EC:7.4.2.8	
**GE001089**	*FliK*	Flagellar hook-length control protein FliK		

Table S9. Effect of strain ZS-3 on aboveground and underground biomass of *C. camphora*

Group	Aboveground		Underground	
	Fresh weight (g)	Dry weight (g)	Fresh weight (g)	Dry weight (g)
S-CK	8.37±0.78b	3.57±0.19c	5.03±0.26b	2.70±0.39b
S-ZS-3	15.69±1.52a	7.34±0.89a	11.2±2.54a	5.73±1.33a
N-CK	9.27±0.67b	3.92±0.45c	5.26±0.64b	3.25±0.24b
N-ZS-3	13.67±1.44a	5.52±0.63b	9.27±0.78a	5.28±0.51a

Note: Results are the mean ± standard deviation from 15 independent experiments. Different lowercase letters above the bars represent significant differences based on one-way ANOVA ($P < 0.05$). The four treatment groups were as follows: (1) S-CK: saline soil inoculated with ddH2O; (2) S-ZS-3: saline soil inoculated with ZS-3; (3) N-CK: neutral soil inoculated with ddH2O; and (4) N-ZS-3 neutral soil inoculated with ZS-3.

Table S10. Effect of strain ZS-3 on root development of *C. camphora*

Group	Total root length/cm	Root surface area/cm^2	Root volume/cm^3	Root tip number/individual	Bifurcation Number/individual
S-CK	113.09±12.56c	173.29±18.28c	19.60±1.31c	2851.67±123b	4182.33±127.97d
S-ZS-3	185.92±10.92a	379.41±31.08a	37.80±2.99a	3398±210.23a	12398.67±1164.30a
N-CK	141.20±10.38b	148.29±10.33c	23.53±3.98c	2878±349.69b	6123±290.10c
N-ZS-3	157.55±9.677b	280.68±22.64b	28.95±2.09b	3126±306.99ab	9226±912.69b

Note: Results are the mean ± standard deviation from 15 independent experiments. Different lowercase letters above the bars represent significant differences based on one-way ANOVA ($P < 0.05$). The four treatment groups were as follows: (1) S-CK: saline soil inoculated with ddH_2O; (2) S-ZS-3: saline soil inoculated with ZS-3; (3) N-CK: neutral soil inoculated with ddH_2O; and (4) N-ZS-3 neutral soil inoculated with ZS-3.
